# Tau inhibits PKA by nuclear proteasome‐dependent PKAR2α elevation with suppressed CREB/GluA1 phosphorylation

**DOI:** 10.1111/acel.13055

**Published:** 2019-10-31

**Authors:** Jinwang Ye, Yaling Yin, Huanhuan Liu, Lin Fang, Xiaoqing Tao, Linyu Wei, Yue Zuo, Ying Yin, Dan Ke, Jian‐Zhi Wang

**Affiliations:** ^1^ Department of Pathophysiology School of Basic Medicine Key Laboratory of Ministry of Education of China for Neurological Disorders Tongji Medical College Huazhong University of Science and Technology Wuhan China; ^2^ Department of Physiology and Neurobiology School of Basic Medical Sciences Xinxiang Medical University Xinxiang China; ^3^ School of Pharmacy Xinxiang Medical University Xinxiang China; ^4^ Department of Physiology School of Basic Medicine Tongji Medical College Huazhong University of Science and Technology Wuhan China; ^5^ Co‐innovation Center of Neurodegeneration Nantong University Nantong China

**Keywords:** CREB, GluA1, PA28γ, PKA, proteasome, synaptic plasticity, Tau

## Abstract

Intraneuronal accumulation of wild‐type tau plays a key role in Alzheimer's disease, while the mechanisms underlying tauopathy and memory impairment remain unclear. Here, we report that overexpressing full‐length wild‐type human tau (hTau) in mouse hippocampus induces learning and memory deficits with remarkably reduced levels of multiple synapse‐ and memory‐associated proteins. Overexpressing hTau inhibits the activity of protein kinase A (PKA) and decreases the phosphorylation level of cAMP‐response element binding protein (CREB), GluA1, and TrkB with reduced BDNF mRNA and protein levels both in vitro and in vivo. Simultaneously, overexpressing hTau increased PKAR2α (an inhibitory subunit of PKA) in nuclear fraction and inactivated proteasome activity. With an increased association of PKAR2α with PA28γ (a nuclear proteasome activator), the formation of PA28γ‐20S proteasome complex remarkably decreased in the nuclear fraction, followed by a reduced interaction of PKAR2α with 20S proteasome. Both downregulating PKAR2α by shRNA and upregulating proteasome by expressing PA28γ rescued hTau‐induced PKA inhibition and CREB dephosphorylation, and upregulating PKA improved hTau‐induced cognitive deficits in mice. Together, these data reveal that intracellular tau accumulation induces synapse and memory impairments by inhibiting PKA/CREB/BDNF/TrkB and PKA/GluA1 signaling, and deficit of PA28γ‐20S proteasome complex formation contributes to PKAR2α elevation and PKA inhibition.

## Graphical abstract

Tau overexpressed decreases the formation of PA28γ‐20S proteasome complex by increasing the association of PKAR2α (an inhibitory subunit of PKA) with PA28γ (a nuclear proteasome activator), which leads to reduced PKAR2α degradation in ubiquitin‐independent proteasome degradation pathway mediated by PA28γ‐20S proteasome complex. PKA inhibition by elevated PKAR2α in nuclear fraction finally induces synapse impairments and memory deficits by inhibiting PKA/CREB/BDNF/TrkB and PKA/GluA1 signaling.

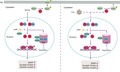

## 1 INTRODUCTION

Intracellular accumulation of the abnormally modified microtubule‐associated protein tau is a hallmark pathology in the brain of Alzheimer's disease (AD) and the other tauopathies. However, how tau accumulation induces synapse and memory deficits is unclear. Tau is a axonal‐located microtubule‐associated protein involving in microtubule assemble and stabilization; however, abnormal dendritic translocation of tau proteins induces the dendritic complex loss and synaptic neurodegeneration (Shentu et al., 2018). The increased total tau in plasma and accumulated tau in brain correlate with the severity of memory deficit in AD (Mielke et al., 2017; Thal et al., 2000). Clustering of tau fibrils in the membrane imbalance neuronal homeostasis by the impaired synaptic composition of Na^+^/K^+^‐ATPase and α‐amino‐3‐hydroxy‐5‐methylisoxazole‐4‐propionic acid (AMPA) receptors (Shrivastava et al., 2019).

Phosphorylation of cAMP‐response element binding protein (CREB) at Ser133 activates the transcription factor and promotes the synthesis of synapse‐ and memory‐associated proteins, thus potentiates learning and memory (Tully, Bourtchouladze, Scott, & Tallman, 2003), while CREB dephosphorylation at Ser133 has been observed in learning and memory disorders. For instance, both Aβ treatment and tau overexpression inhibit CREB phosphorylation with learning and memory deficits (Yin et al., 2016). As a transcription factor, CREB‐mediated transcription of BDNF is sufficient to promote neuronal dendrite outgrowth, enhance synapse formation, and improve learning and memory (Bu et al., 2017; Segarra‐Mondejar et al., 2018). CREB also mediates the transcription of AMPA receptors, which is the molecular basis of excitatory synaptic transmission (Middei et al., 2013).

Protein kinase A (PKA) is the most recognized kinase in phosphorylating CREB at Ser133 and AMPA‐type glutamate receptor trafficking, by which it promotes transcription of synaptic proteins and plays a crucial role in learning and memory formation (Taylor, Ilouz, Zhang, & Kornev, 2012). β‐amyloid (Aβ) can inhibit PKA (Tong, Thornton, Balazs, & Cotman, 2001), while activating PKA enhances long‐term potentiation (LTP) and memory formation (Xie et al., 2016). PKA activation leads to fast exocytosis of AMPA receptors through GluA1 phosphorylation at Ser845, which in turn potentiates LTP (Oh, Derkach, Guire, & Soderling, 2006). PKA is a tetrameric protein consisting of two regulatory subunits (PKA‐Rs) and three catalytic subunits (PKA‐Cs). When in an inactive state, the pseudo‐substrate sequence of the PKA‐Rs blocks the active site on the PKA‐Cs. Three PKA‐Cs (C‐α, C‐β, and C‐γ) and two PKA‐Rs (RI and RII) families have been discovered, and all PKA‐Rs have different cAMP binding properties (Ilouz et al., 2017). The two R families have two isoforms: α and β (RI‐α, RI‐β, RII‐α, and RII‐β). When cAMP binds to the R subunit, it can alleviate the self‐inhibiting contact and release activated monomeric PKA‐Rs (Skalhegg & Tasken, 2000). PKA‐Rs released are immediately degraded by proteasome to maintain the activated state of PKA‐Cs (Tai & Schuman, 2008). The liberated PKA‐Cs are required to sustain PKA function.

Aggregation of misfolded proteins, such as tau, α‐synuclein, and huntingtin, compromises the function of 26S proteasome complex, leading neurons susceptible to protein homeostasis disorder, thereby contributing to the pathologies of neurodegeneration (Myeku & Duff, 2018). The canonical proteasome consists of two distinct subcomplexes, a 19S regulatory particle (RP) and a 20S catalytic particle (CP; Livneh, Cohen‐Kaplan, Cohen‐Rosenzweig, Avni, & Ciechanover, 2016). The 19S RP binds to the 20S CP to form the 26S proteasome that specially recognizes and degrades polyubiquitylated proteins (Budenholzer, Cheng, Li, & Hochstrasser, 2017), which is referred as the ubiquitin‐dependent degradation. PKA phosphorylates 19S RP Rpn6 to promote the ubiquitin‐dependent proteasome degradation process (Lokireddy, Kukushkin, & Goldberg, 2015). PA28γ is a nuclear activator of the 20S proteasome, and formation of PA28γ‐20S proteasome complex mediates ubiquitin‐independent degradation (Jonik‐Nowak et al., 2018). Intracellular tau accumulation results in proteasome inhibition (Keck, Nitsch, Grune, & Ullrich, 2003), and activation of cAMP‐PKA signaling prevents tau‐related 26S proteasome impairment and cognitive dysfunction (Myeku et al., 2016). However, whether PA28γ‐20S ubiquitin‐independent degradation participates in tau pathology remains unknown.

Pan‐neuronal overexpression of tau induces synapse and memory deficits in mice, and simultaneously, a remarkable CREB dephosphorylation was shown (Yin et al., 2016). However, it is not known how tau accumulation downregulates CREB phosphorylation. By neuron‐specific overexpression of human full‐length wild‐type tau (hTau), we demonstrate in the present study that hTau accumulation inhibits PKA/CREB signaling with downregulation of multiple synapse‐ and memory‐associated proteins and GluA1‐containing AMPARs surface expression both in vitro and in vivo. The mechanism underlying PKA inactivation involves an impaired nuclear proteasome‐mediated PKAR2α elevation. Simultaneous downregulating PKAR2α or upregulating proteasome restored PKA activity, CREB and GluA1 phosphorylation, BDNF expression with increased spine density, dendritic complex, and membranous trafficking of GluA1. Activating PKA/CREB signaling also rescued learning and memory deficits in hTau‐overexpressing mouse model.

## 2 RESULTS

### 2.1 Overexpressing hTau in mouse hippocampal CA3 neurons induces memory deficits with PKA inhibition and CREB/GluA1 dephosphorylation

**Figure 1:**
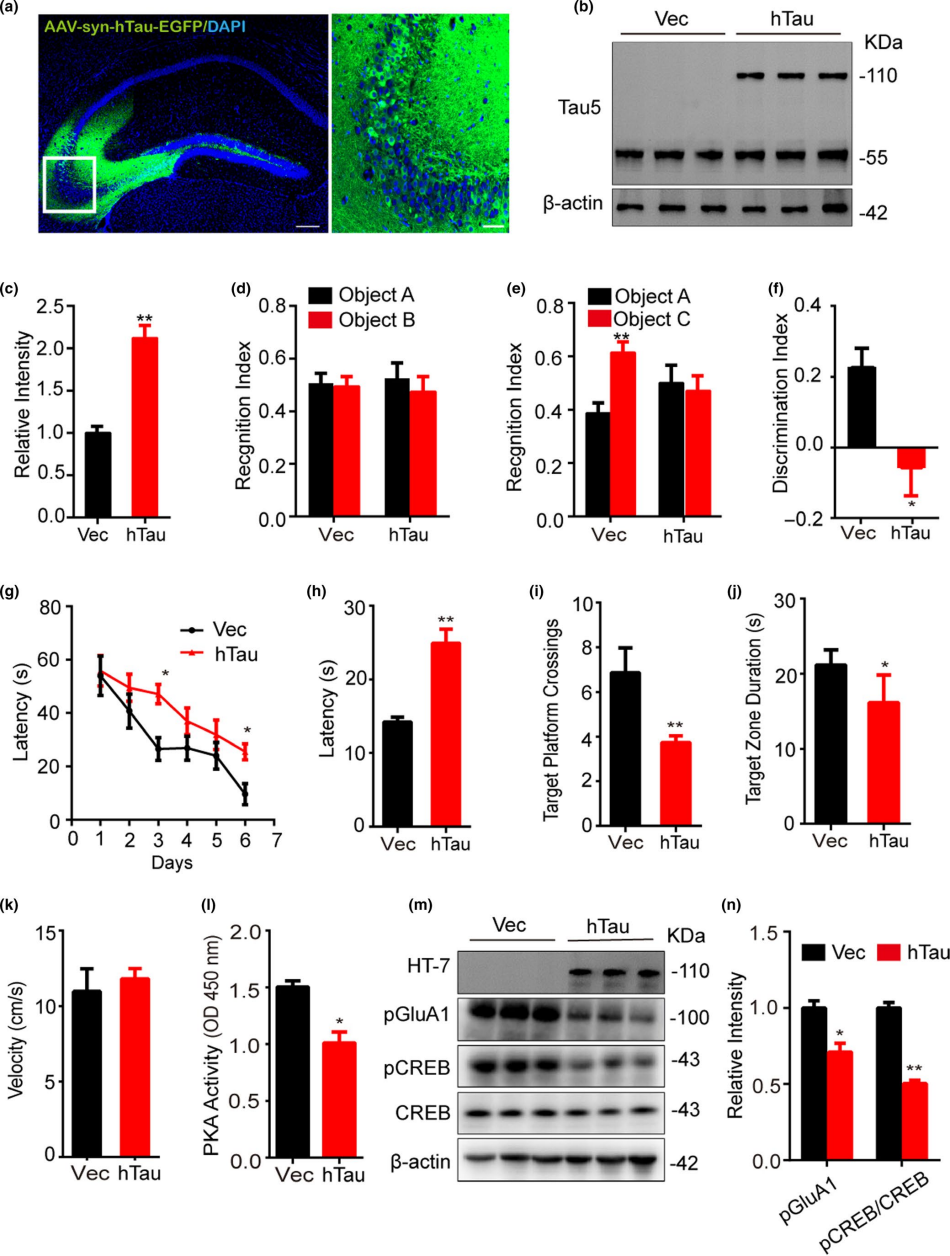
Overexpressing hTau in mouse hippocampal CA3 neurons induces memory deficits with PKA inhibition and CREB/GluA1 dephosphorylation. (a–c) AAV‐syn‐hTau‐EGFP was infused into the hippocampal CA3 subset of 2‐month‐old mice, and after one month, the expression of hTau in the infused site (scale bar: left, 250 μm; right, 50 μm) was confirmed by direct fluorescence imaging (a), and Western blotting (110 kDa) using Tau5 (b and c; *n* = 6 mice per group). (d–f) Overexpressing hTau induces memory deficit measured by novel object recognition (NOR) test. The recognition index measured at the first training day (d), and the reduced recognition index (e) and the discrimination index (f) recorded at 24 hr after training (*n* = 15 mice per group) were analyzed. (g–k) Overexpressing hTau induces spatial learning and memory deficits measured by Morris water maze (MWM). The spatial learning deficit was shown by the increased latency to find the hidden platform during 6 days of training (g), while the memory deficit was shown by the increased latency to reach the platform site (h), decreased target platform crossings (i), and decreased time spent in the target zone (j) measured at day 8 by removing the platform (*n* = 15 mice per group). The swimming speed was not changed (k). (l) Overexpressing hTau inhibits PKA activity measured by using a PKA activity assay kit. The injected hippocampal CA3 subsets were dissected, and the tissue extracts were used. (m, n) Overexpressing hTau reduced levels of pGluA1 and pCREB measured by Western blotting (m) and the quantification (n); HT7 specifically reacts with human tau. Data were expressed as mean ± *SEM*, *p < .05, **p < .01 versus Vec, but *p < .05 versus object A in (e)

To explore the in vivo toxicity of neuronal tau accumulation, we made neuron‐specific overexpression of hTau by stereotaxic infusion of AAV‐syn‐hTau‐EGFP (hTau) into the mouse hippocampal CA3 subset, and the same volume of AAV‐syn‐EGFP (Vec) was infused as control. After one month, robust expression of AAV‐syn‐hTau‐EGFP was confirmed by direct fluorescence imaging and Tau5 staining (Figure 1a,b). On average, the total tau level increased to ~2.1‐fold of the control (Figure 1c), which may represent an early Braak stage of tau pathology in the AD patients according to the previous reports (Kurbatskaya et al., 2016). The oligomeric and fibrillary tau proteins were detected by using an anti‐tau oligomer antibody T22 (Figure S1a) and Thioflavin‐S staining (Figure S1b), respectively. Then, the effects of hTau accumulation on learning and memory of mice were measured by novel object recognition (NOR) and Morris water maze (MWM) test. In NOR test, the recognition index was comparable between hTau and the control groups in the training trial (Figure 1d). However, the recognition and discrimination indexes to the new object were significantly decreased in the hTau mice during test trials measured at 24 hr after the training trial (Figure 1e,f), indicating an impaired long‐term memory. In MWM test, we observed that neuronal overexpression of hTau in hippocampal CA3 subset for one month induced spatial learning deficit during 6 days of training trial (Figure 1g). The spatial memory was tested on day 8 by removed the platform. We observed that the hTau‐overexpressing mice exhibited memory deficits shown by an increased latency to find the platform (Figure 1h), decreased target platform crossings (Figure 1i), and target zone duration (Figure 1j). No difference in swimming speed was detected between hTau and the control mice (Figure 1k). We also measured PKA activity and the PKA‐dependent phosphorylation of the synapse‐ and memory‐associated proteins by dissecting hippocampal CA3 subsets under a fluorescence microscope. The results showed that PKA activity was remarkably inhibited in hTau‐infused CA3 extracts compared with the AAV‐vector infused controls (Figure 1l); simultaneously, the phosphorylation level of pS133‐CREB and pS845‐GluA1 was decreased (Figure 1m,n). These in vivo data validate the involvement of PKA inhibition in hTau‐induced synapse and memory deficits.

### 2.2 Overexpressing hTau in cultured hippocampal neurons decreases synaptic proteins with PKA inhibition and CREB/TrkB and GluA1 dephosphorylation

**Figure 2:**
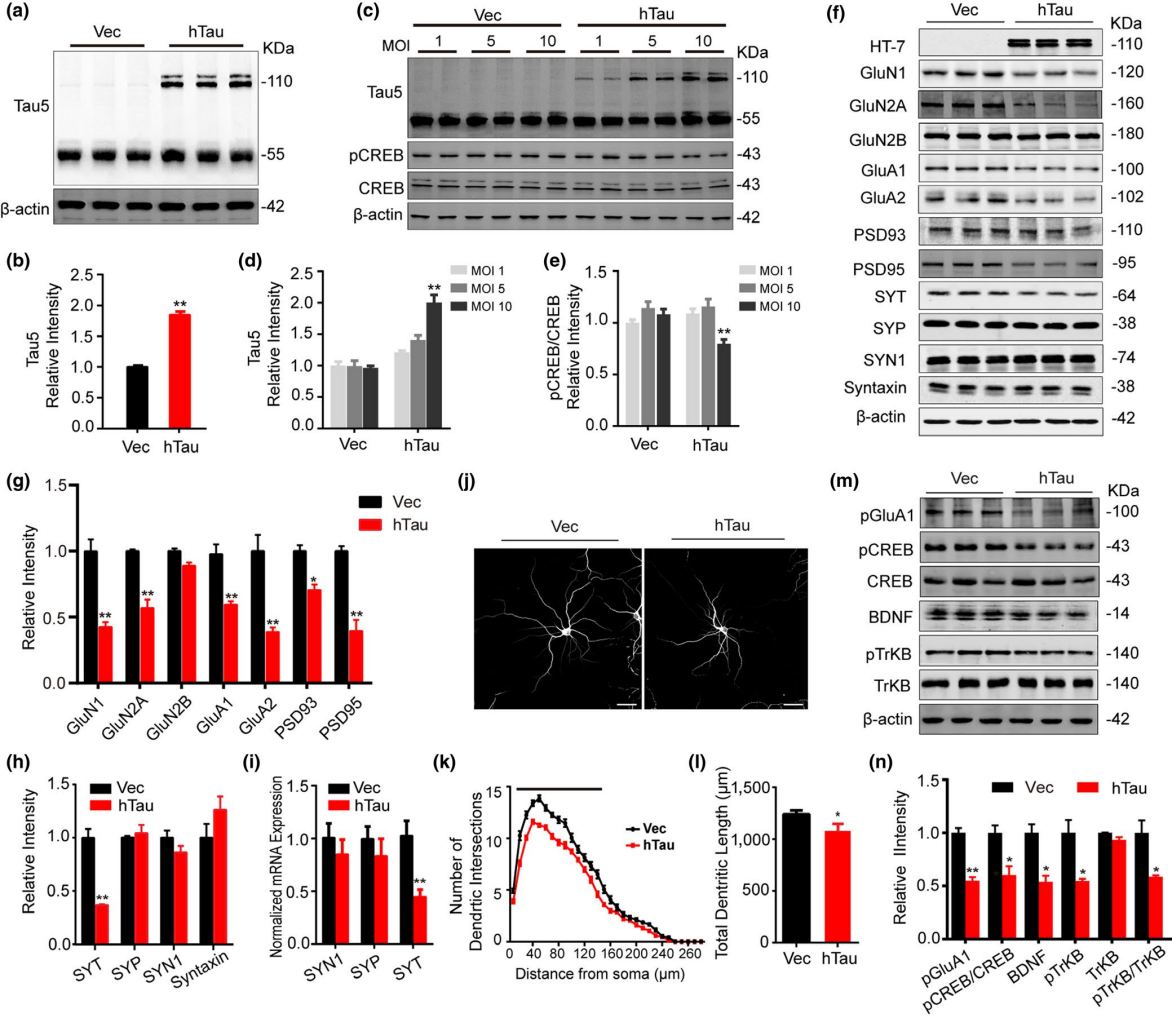
Overexpressing hTau in primary cultured neurons decreases synaptic proteins with reduced phosphorylation of CREB/TrkB and GluA1. (a, b) The hippocampal neurons (5 *div*) were infected with lenti‐syn‐hTau‐mCherry or the empty vector and cultured for another 7 days, and the expression of hTau (110 kDa) was confirmed by Western blotting using Tau5 (reacts with total tau). (c, d) Infection of different MOI lenti‐hTau virus induced a dose‐dependently increased expression of tau in cultured hippocampal neurons measured by Western blotting using Tau5 (*n* = 6, **p < .01 vs. MOI 1). (e) Infection of 10 MOI lenti‐hTau virus significantly decreased CREB phosphorylation (*n* = 6, **p < .01 vs. MOI 1). (f–h) Overexpressing hTau induces reduction of multiple synaptic proteins. The hippocampal neurons (5 *div*) were infected with lenti‐syn‐hTau‐mCherry or the empty vector and cultured for another 7 days, and the levels of presynaptic (g) and postsynaptic (h) proteins were measured by Western blotting. The experiments were repeated three times from nine different batches of primary cultured neurons. (i) Overexpressing hTau decreases mRNA level of SYT with no significant effect on SYN1 and SYP measured by qPCR. (j–l) Overexpressing hTau decreases dendrites branches and total dendrite length. The cultured hippocampal neurons (18 *div*) were stained with MAP2, and then, the dendrite arborizations and total length were analyzed by Sholl (scale bar: 50 μm). The total dendrite length and the branches at 20 to 140 μm from soma were measured (*n* = 30 per group from six independent cultures, black line, *p* < .05, vs. Vec). (m, n) Overexpressing hTau decreases CREB/TrkB/GluA1 phosphorylation and reduces BDNF measured by Western blotting. The phosphorylation levels of CREB, TrkB, and GluA1 were normalized to the total protein levels or to β‐actin. The experiments were repeated three times from nine different batches of primary neurons. Data were expressed as mean or mean ± *SEM*, *p < .05, **p < .01 versus Vec, but **p < .01 versus MOI 1 in (d) and (e)

To explore the mechanisms underlying hTau‐related neurodegeneration, we overexpressed human tau (lenti‐hTau‐mCherry) or the empty vector (lenti‐mCherry) in cultured hippocampal neurons (5 *div*). After 7 days, the overexpression of tau was confirmed by Western blotting using Tau5, and the total tau level increased to ~1.8‐fold of the control level (Figure 2a,b). Furthermore, infection of different multiplicity of infection (MOI) virus induced a dose‐dependent increase in total tau expression (Figure 1c,d). As only infection of 10 MOI virus induced significant reduction of CREB phosphorylation (Figure 1e), we used 10 MOI for the rest studies on the expression and phosphorylation levels of multiple synapse‐ and memory‐associated proteins. Overexpressing hTau decreased levels of NMDARs (GluN1 and GluN2A), AMPARs (GluA1 and GluA2), postsynaptic density proteins (PSD93 and PSD95), and synaptotagmin (SYT), without affecting GluN2B, synaptophysin (SYP), synapsin I (SYN1), and syntaxin (Figure 2f–h). Overexpressing hTau also decreased mRNA level of SYT without significant effect on the mRNA levels of SYN1 and SYP (Figure 2i). The dendritic length and complexity were reduced in hTau‐overexpressing neurons (Figure 2j–l). The activity of PKA was inhibited, and the levels of phosphorylated CREB at Ser133 (pCREB), phosphorylated GluA1 at Ser845 (pGluA1), the phosphorylated TrkB at Tyr706 (pTrkB), and the protein level of BDNF were all decreased after overexpressing hTau (Figure 2m,n). These data suggest that overexpressing hTau induces synapse impairment with the mechanisms involving inhibition of PKA/CREB/BDNF signaling.

### 2.3 Upregulating PKA rescues hTau‐induced CREB/GluA1 dephosphorylation and BDNF expression with improved synaptic plasticity and cognitive functions

**Figure 3:**
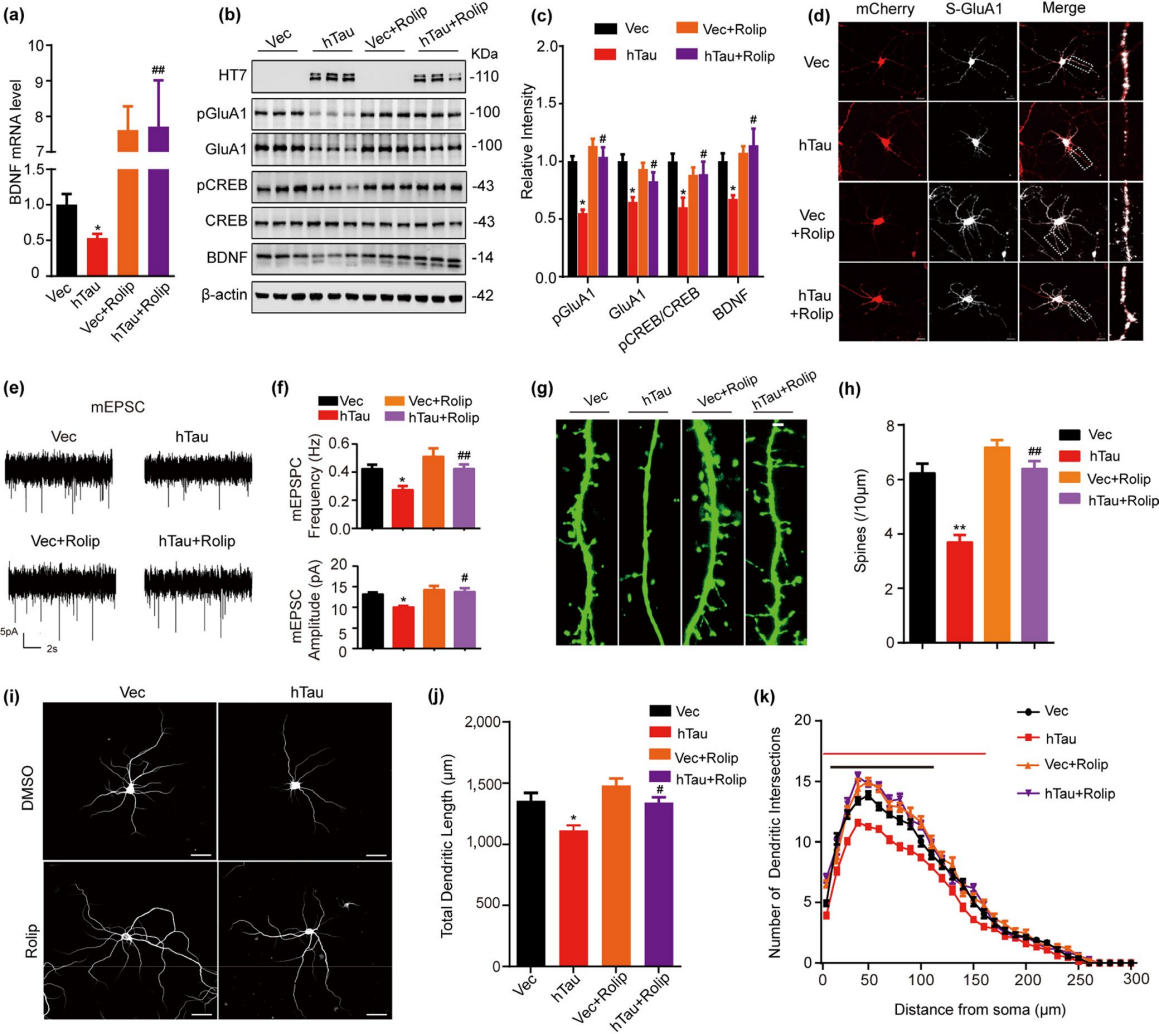
Upregulating PKA rescues hTau‐induced synaptic impairments with increased CREB/GluA1 phosphorylation and BDNF expression. (a–c) The hippocampal neurons (5 *div*) were infected with lenti‐syn‐hTau‐mCherry or the empty vector for 7 days, and then, the neurons were treated with 40 μM rolipram to activate PKA for 12 hr. The mRNA level of BDNF was measured by qPCR (a); the protein levels of pGluA1, GluA1, pCREB, CREB, and BDNF were detected by Western blotting (b); and the quantitative analysis was performed (c) (*n* = 9 per group). (d) Upregulating PKA restores the hTau‐suppressed membrane localization of GluA1 measured by incubation the living neurons (12 *div*) with an extracellular N‐terminal of GluA1 antibody (scale bar, 20 μm). (e, f) Upregulating PKA restores the hTau‐suppressed synaptic transmission. The representative traces of mEPSCs recorded by whole‐cell patch clamp in hTau‐ or Vec‐infected primary hippocampal neurons (15 *div*) treated with rolipram or vehicle. Scale bars: 5 pA, 2s (e). The average frequency and amplitude of mEPSCs were collected from at least 7–12 neurons per group (f). (g, h) Upregulating PKA restores the hTau‐induced spine impairments. For spine analysis in vitro, the hippocampal neurons (12 *div*) were infected with lenti‐EGFP and lenti‐syn‐hTau‐mCherry or the control vector. Spine density (21 *div*) was measured by two‐photon confocal laser scanning microscopy (g). The spine number per 10 μm of dendritic length was analyzed using ImageJ software (h). At least 30 neurons from six independent cultures were collected for each group. Scale bar 2 μm. (i) Upregulating PKA restores the hTau‐suppressed dendrite development in cultured hippocampal neurons (18 *div*) stained with MAP2. (j) The total length and arborizations of dendrites were analyzed by Sholl (scale bar: 50 μm). At least 30 neurons from six independent cultures were analyzed for each group. (k) Upregulating PKA rescues hTau‐induced the decrease dendrites branches at 10 to 160 μm from soma (black line, *p* < .05, vs. Vec, red line, *p* < .05 vs. hTau). At least 30 neurons from six independent cultures were analyzed for each group. Data were expressed as mean or mean ± *SEM*, *p < .05, **p < .01 versus Vec. **^#^** *p* < .05, **^##^** *p* < .01 versus hTau

To verify the role of PKA in hTau‐induced inhibition of PKA/CREB/BDNF signaling and the impairments of synaptic and cognitive functions, we first infected primary cultured hippocampal neurons (5 *div*) with lenti‐syn‐hTau‐mCherry or lenti‐syn‐mCherry for 7 days and treated the neurons with rolipram, a PKA agonist for 12 hr. Then, the activity of PKA/CREB/BDNF signaling and synaptic plasticity was measured. Rolipram treatment attenuated hTau‐induced PKA inhibition with restoration of pS133‐CREB, pS845‐GluA1, BDNF mRNA, and protein levels (Figure 3a–c). Simultaneously, the expression of surface GluA1, which plays an important role in synaptic transmission and is Ser845‐phosphorylation‐dependent (Oh et al., 2006), was remarkably suppressed in hTau‐overexpressing neurons, while rolipram treatment restored the surface GluA1 level measured in living neurons with an extracellular GluA1 antibody (Figure 3d). Whole‐cell patch‐clamp recordings were also performed to detect the synaptic transmission in primary cultured hippocampal neurons. Overexpressing hTau decreased both frequency and the amplitude of mEPSC, which were restored by rolipram (Figure 3e,f). The spine density, dendrite length, and complexity were decreased by overexpressing hTau and rolipram rescued the dendritic impairments in cultured hippocampal neurons (Figure 3g–k). These in vitro data confirm that PKA inhibition plays a crucial role in hTau‐induced synapse impairments.

**Figure 4:**
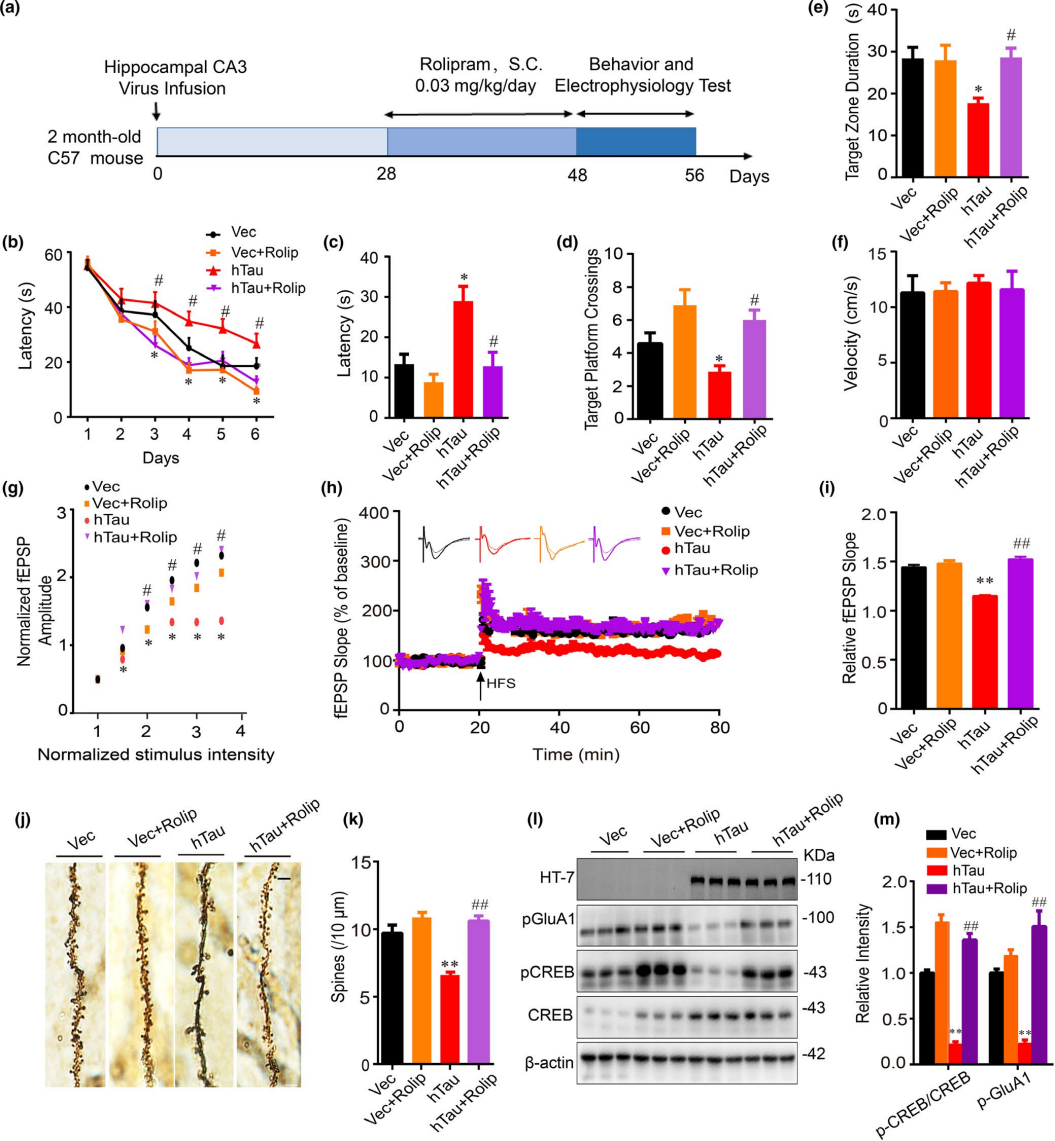
Upregulating PKA rescues synaptic dysfunction and cognitive decline with restoration of CREB/GluA1 phosphorylation. (a) Schematics show the treatments of mice. AAV‐syn‐hTau‐EGFP or the empty vector was infused stereotaxically into the hippocampal CA3 of 2‐month‐old mice for one month, and then, rolipram (PKA activator, 0.03 mg/kg per day) or normal saline (NS) was infused intraperitoneally for 20 days. After MWM test, the mice were sacrificed and the hippocampi were dissected for electrophysiology recording and Western blotting analysis. (b–f) Simultaneous activation of PKA attenuates hTau‐induced spatial learning and memory deficits shown by the reduced latency to find the hidden platform during 6 days of training on MWM (b), and the decreased latency to find the platform site (c), the increased target platform crossing (d), and the duration in the target zone measured at day 8 after removed the platform (e). The swimming velocity was not changed (f) (*n* = 15 mice per group). (g–i) Simultaneous activation of PKA rescues hTau‐induced inhibition of LTP induction shown by the restored fEPSP amplitudes (input–output curve) induced by minimum stimulation intensity (g), and the restored fEPSP slope induced by applying three trains of high‐frequency stimulation (HFS) (h, i). (j, k) Simultaneous activation of PKA rescues hTau‐induced spine impairments. The mice were infected and treated as described in panel a. Golgi staining was used to evaluate the changes of dendritic spines in the hippocampal CA3 subset of the mice, and at least 30 neurons from six mice per group were analyzed. (l, m) Simultaneous activation of PKA rescues hTau‐induced dephosphorylation of GluA1 and CREB measured by Western blotting. HT7 specifically reacts with human tau. The data were expressed as mean ± *SEM*. *p < .05, **p < .01 versus Vec, ^#^ *p* < .05, ^##^ *p* < .01, versus hTau

To validate whether activating PKA could improve cognitive deficits in mice, dorsal hippocampal CA3 of 2‐month‐old mice was stereotaxically infused with AAV‐syn‐hTau‐EGFP or AAV‐syn‐EGFP as vector control. After four weeks, the mice were intraperitoneally infused with rolipram (0.03 mg/kg per day) or normal saline (NS) for 20 days and then learning and memory were tested (Figure 4a). After rolipram treatment, MWM test was performed to test the spatial learning and memory. The mice with hTau overexpression displayed spatial learning and memory deficits, while simultaneous activating PKA by rolipram treatment attenuated hTau‐induced learning and memory deficits (Figure 4b–e). No difference in swimming speed was shown among the four groups (Figure 4f). By using electrophysiological recordings, we observed a pronounced inhibition of LTP induction in hTau‐overexpressing mice compared with the control group, and this inhibition was attenuated by rolipram (Figure 4g–i). By Golgi staining in brain slices, we also observed that rolipram treatment restored hTau‐induced reduction of spine density (Figure 4j,k) with attenuation of CREB and GluA1 phosphorylation (Figure 4l,m). These in vivo data further confirm that PKA inhibition plays a crucial role in hTau‐induced synapse impairment and memory deficit.

### 2.4 Overexpressing hTau increases nuclear PKAR2α and inhibits proteasome, the latter contributes to the hTau‐induced PKAR2α elevation

**Figure 5:**
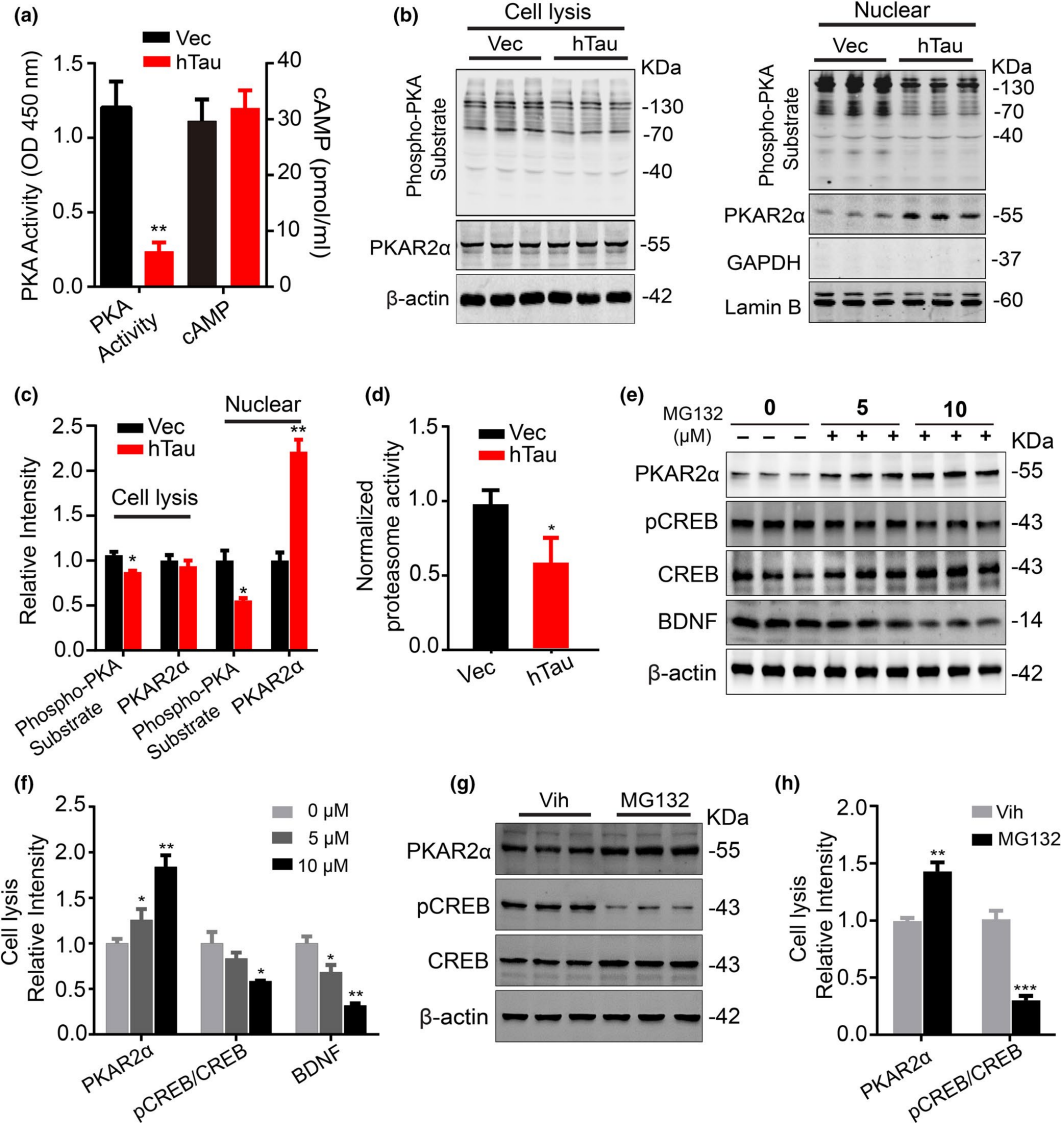
Overexpressing hTau inactivates PKA with proteasome‐involved elevation of PKAR2α and inhibition of CREB/BDNF signaling. (a) Overexpressing hTau inhibits PKA without changing cAMP in cultured hippocampal neurons (5 + 7 *div*) measured, respectively, by using PKA activity assay kit and the cAMP ELISA kit. (b, c) Overexpressing hTau inhibits PKA activity with an elevated PKAR2α in nuclear fraction of the cultured neurons. The hippocampal neurons (5 *div*) were infected with lenti‐syn‐hTau‐mCherry or the empty vector for 7 days, and then, PKA activity (using the phosphor‐PKA substrate) and the level of PKAR2α (PKA regulatory subunit) in total cell lysis and the nuclear fraction were measured by Western blotting. (d) Overexpressing hTau inhibits proteasome activity. The hippocampal neuron (5 *div*) was infected with lenti‐syn‐hTau‐mCherry or the empty vector for 7 days, and the proteasome activity in the cell lysis was measured using a proteasome activity kit. (e, f) Inhibiting proteasome by treating N2a cells with different concentrations of MG132 induces PKAR2α elevation with reduced CREB phosphorylation and BDNF expression measured by Western blotting. (g, h) Inhibiting proteasome by treating cultured hippocampal neurons with MG132 (10 μM) for 12 hr induces PKAR2α elevation with reduced CREB phosphorylation and BDNF expression measured by Western blotting. Data were expressed as mean ± *SEM*, *p < .05, **p < .01 versus Vec, but *p < .05, **p < .01 versus 0 μM in (f), **p < .01, ***p < .001 versus Vih in (h). The experiments were carried out from nine different batches of N2a cells and cultured hippocampal neurons

To explore the mechanism underlying the hTau‐induced PKA inhibition, we measured different subunits of PKA by infecting the primary hippocampal neurons (5 *div*) with lenti‐syn‐hTau‐mCherry or lenti‐syn‐mCherry viruses for 7 days. The reduced PKA activity in hTau‐overexpressing neurons was detected using a PKA activity assay kit (Figure 5a). By Western blotting using PKA substrate antibody, the reduced PKA activity in total extracts and the nuclear fraction in hTau‐overexpressing neurons were also detected (Figure 5b,c). The catalytic subunits of PKA (PKA‐Cs) are regulated by the regulatory subunits (PKA‐Rs) and cAMP, when cAMP binds to the PKA‐Rs, PKA‐Rs can be removed from PKA‐Cs, and activated monomeric PKA‐Cs are released (Skalhegg & Tasken, 2000; Taylor et al., 2012). Therefore, we first measured cAMP level but no significant difference was shown between hTau and control groups (Figure 5a), suggesting that hTau‐induced PKA inhibition is not cAMP‐dependent. Then, we measured PKA‐Rs and PKA‐Cs in total extracts and nuclear fractions. Overexpressing hTau increased nuclear level of PKAR2α without changing PKAR1α, PKAR1β, PKAR2β, and PKA‐Cs in both fractions (Figure 5b,c, S2a–c). Significant elevation of PKAR2α in nuclear fraction was also detected in brain slices after hTau overexpression (Figure 2d). These data suggest that hTau accumulation may inhibit PKA through upregulating nuclear PKAR2α.

To further explore the mechanisms underlying PKAR2α elevation, we measured the proteasome activity. With the upregulated PKAR2α protein level in total extract and the nuclear fraction (Figure 5b,c), a remarkably inhibited proteasome activity was detected in hTau‐overexpressing cells compared with the controls (Figure 5d). Furthermore, inhibition of proteasome by treating N2a cells with different concentrations of MG132 for 12 hr induced a concentration‐dependent increase in PKAR2α with reduced CREB phosphorylation at Ser133 and BDNF expression (Figure 5e,f). Treating the cultured hippocampal neurons with 10 μM MG132 also induced the same changes as observed in N2a cells (Figure 5g,h). These data indicate that proteasome inhibition at least contributes to the hTau‐induced nuclear elevation of PKAR2α, which in turn inhibits PKA/CREB/BDNF signaling.

### 2.5 Overexpressing hTau disrupts 20S‐PA28γ complex formation and upregulating PA28γ or downregulating PKAR2α rescues PKA/CREB signaling

**Figure 6:**
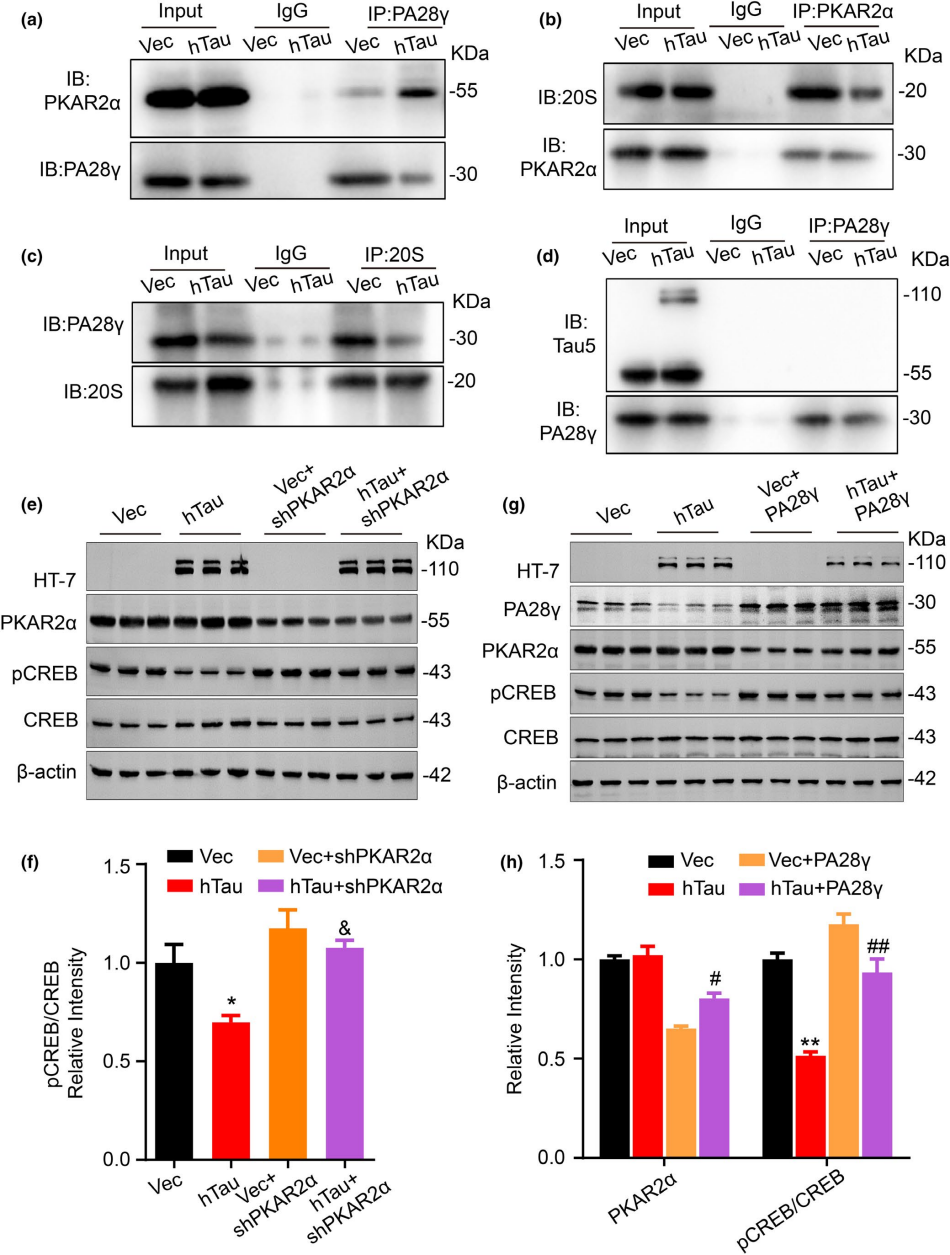
Overexpressing hTau disrupts PA28γ‐20S proteasome complex formation and downregulating PKAR2α or upregulating PA28γ attenuates hTau‐induced PKA/CREB inhibition. (a) Overexpressing hTau increases association of PKAR2α with PA28γ. The cultured hippocampal neurons infected with lenti‐syn‐hTau‐mCherry virus for 7 days, and PA28γ was co‐immunoprecipitated by anti‐PA28γ antibody and detected by Western blotting using anti‐PKAR2α and anti‐PA28γ antibody. For all co‐immunoprecipitations, results are received from three independent experiments. (b) Overexpressing hTau decreases association of PKAR2α with 20S proteasome subunit. PKAR2α was co‐immunoprecipitated using anti‐PKAR2α antibody and detected by Western blotting using anti‐20S proteasome subunit and anti‐PKAR2α antibody. (c) Overexpressing hTau decreases association of PA28γ with 20S proteasome subunit. 20S proteasome subunit was co‐immunoprecipitated using anti‐20S protease subunit and detected by Western blotting using anti‐PA28γ antibody and anti‐20S proteasome subunit. (d) Tau dose not bind to PA28γ. The cultured hippocampal neuron extract was co‐immunoprecipitated using anti‐PA28γ, and tau was not detected in the precipitates by Tau5. (e, f) Downregulating PKAR2α by shRNA attenuates hTau‐induced CREB dephosphorylation. The primary cultured hippocampal neurons (*5 div*) were co‐infected with hTau and shPKAR2α lentivirus. After 7 days, the indicated proteins were measured by Western blotting. (g, h) Upregulating PA28γ attenuates hTau‐induced PKAR2α elevation and CREB dephosphorylation. The hTau and PA28γ lentivirus were co‐infected in primary cultured hippocampal neurons (*5 div)* for 7 days, and the indicated proteins were measured by Western blotting. Data were expressed as mean ± *SEM*. For Western blotting, the n number was 9 (from nine different batches of cultured hippocampal neurons). *p < .05, **p < .01, versus Vec, ^&^ *p* < .05, versus hTau in (f), ^#^ *p* < .05, ^##^ *p* < .01, versus hTau in (h)

Inhibition of proteasome activity has been widely observed in AD and other tauopathy, and PKA‐Rs are degraded in 26S proteasome (Tai & Schuman, 2008). As the hTau‐induced PKAR2α elevation was largely detected in the nuclei, we measured whether PA28γ (a nuclear activator of the 20S proteasome)‐mediated ubiquitin‐independent degradation is involved in the hTau‐induced PKAR2α elevation. Overexpressing hTau increased PKAR2α‐PA28γ association and reduced PA28γ‐20S complex formation with decreased PKAR2α‐20S subunit interaction (Figure 6a–c), while the interaction of tau with PA28γ was not detected (Figure 6d). These data indicate that hTau accumulation may indirectly inhibit the ubiquitin‐independent proteasome degradation of PKAR2α and thus inhibit PKA. In hTau‐overexpressing N2a cells or primary neurons, simultaneous downregulating PKAR2α with siRNA or shRNA attenuated hTau‐induced reduction of CREB phosphorylation (Figure 6e,f, S3a,b). The level of PA28γ was decreased in hTau‐overexpressing cells, and upregulating PA28γ attenuated hTau‐induced PKAR2α elevation and CREB dephosphorylation (Figure 6g,h, S3c,d). These data suggest that an impaired 20S‐PA28γ proteasome complex formation may underlie the hTau‐induced PKAR2α upregulation and PKA inhibition, and downregulating PKAR2α or upregulating proteasome rescues PKA/CREB signaling.

## 3 DISCUSSION

Intracellular accumulation of hyperphosphorylated tau has been widely observed in neurodegenerative diseases, such as AD, progressive supranuclear palsy (PSP), corticobasal degeneration, Pick disease, Huntington disease, and frontotemporal dementia with parkinsonism‐17 (Wang & Mandelkow, 2016). Studies also show that tau accumulation induces synaptic dysfunction, neuron loss, and memory impairment in different mouse models (Fu et al., 2017). In AD, it has emerged that Aβ toxicity depends on dendritic function of tau and tau knockout mitigates Aβ toxicity (Ittner et al., 2010). PHF‐tau (paired helical filament) pathology is correlated with the degree of dementia as defined by the clinical dementia rating scale (Thal et al., 2000). In sporadic AD, accumulation of wild‐type tau is the major component of neurofibrillary tangles.

In AD patients, tau pathology was first appeared in the entorhinal cortex, then spread to limbic region of the hippocampus and finally to the whole neocortex. CA3 is one of the critical subset of hippocampal tri‐synaptic circuit, which plays a crucial role in spatial memory. In our previous studies, we consistently observed that CA3 is the most sensitive subset for accumulation of the phosphorylated tau under stresses compared with other subsets of the hippocampus (Li et al., 2016; Yin et al., 2016). To explore the mechanism of tauopathy in AD, we overexpressed the full‐length wild‐type human tau (hTau) specifically in neurons. We found that overexpressing hTau in hippocampal CA3 disrupts spatial learning and memory with inhibition of PKA and decrease in CREB phosphorylation, as well as an impaired proteasome activity. It is well known that synaptic plasticity plays a critical role in memory formation. Synaptic proteins in presynaptic and postsynaptic membrane are the basis of synaptic plasticity. Neurotrophic factor (e.g., BDNF) promotes synaptic formation and dendrite outgrowth. CREB is one of the pivotal transcription factors regulating the expression of various synaptic proteins. Studies show that extracellular application of tau oligomers or direct overexpression of tau or hyperphosphorylation and accumulation of tau by various molecules all reduced synaptic protein expression and disrupted glutamate receptor trafficking (Hoover et al., 2010; Puzzo et al., 2017; Vagnozzi, Giannopoulos, & Pratico, 2018). By overexpression hTau in primary hippocampal neurons, the decreased protein levels of BDNF, GluN1, GluN2A, GluA1, GluA2, PSD93, PSD95, and SYT were also detected with unchanged GluN2B, SYP, and SYN1. Decreased GluA1 phosphorylation at Ser845 also contributes to hTau‐induced synaptic transmission efficacy. Therefore, we speculate that hTau may decrease synaptic protein *via* PKA/CREB‐mediated transcription.

We also noticed that overexpressing hTau only selectively decreased mRNA and protein levels of SYT among several vesicles release‐related presynaptic proteins (including SYN1, SYP and SYT). SYN1, located in the presynaptic terminal, controls the vesicle storage and mobilization (Cesca, Baldelli, Valtorta, & Benfenati, 2010). SYP is a membrane protein located mainly on the presynaptic vesicles with the major functions in regulating vesicle formation and release (Valtorta, Pennuto, Bonanomi, & Benfenati, 2004). SYT is a calcium sensor located on the membrane of presynaptic vesicles with the functions in calcium‐dependent release of vesicles (Jahn & Fasshauer, 2012; Kochubey, Lou, & Schneggenburger, 2011). By website searching, we learn that all these three molecules have the motifs for CREB binding. Thus, the hTau‐induced reduction of SYT may be independent of CREB. Previous study demonstrated that overexpressing hTau decreased sEPSC with a disrupted calcium homeostasis (Yin et al., 2016), suggesting that hTau impairs calcium‐related presynaptic release. As a calcium sensor, SYT may be more vulnerable to the hTau‐induced impairments.

PKA is a crucial kinase in phosphorylating several learning and memory‐associated proteins, such as CREB at Ser133 and GluA1 at Ser845 (Oh et al., 2006; Shen et al., 2016). Here, we observed that overexpression of hTau in hippocampal neurons pronouncedly impaired PKA signaling in both total lysates and the nuclear fraction compared with the vector control. The role of PKA on memory maintenance in different brain subset and pathologies is not always consistent, some researchers show that Aβ inhibits PKA, while activating PKA in hippocampus can improve cognitive functions (Gong et al., 2004; You et al., 2018). Other studies show that PKA accelerates tau phosphorylation, while inhibiting PKA in the prefrontal cortex improves memory in both aged rats and monkeys (Wang et al., 2018; Xie et al., 2016). We observe that rolipram restores memory and LTP deficits in hTau infusion mice. PKA activation by rolipram also rescues CREB/GluA1 phosphorylation with BDNF level and an enhanced surface GluA1 expression, and the latter is Ser845‐phosphorylation‐dependent and plays an important role in synaptic transmission (Oh et al., 2006). Intriguingly, activating PKA by rolipram increased mRNA levels of BDNF with no significant change of pCREB in total cell extracts. A previous study showed that overexpressing hTau increased pCREB in the nuclear fraction (Yin et al., 2016), and the latter is the active pool of CREB binding to BDNF promoter region and thus to promote BDNF expression. Additionally, the pCREB can recruit CREB‐binding proteins, such as C/EBP, which can also regulate expression of BDNF (Alberini & Chen, 2012; Hayes, Towner, & Isackson, 1997). Furthermore, the recovered dendritic length and complexity are also seen after rolipram treatment. These data indicate that PKA may serve as a potential target of tauopathies.

PKA activity is achieved through liberated PKA‐Cs when PKA‐Rs are dissociated from PKA‐Cs. PKA‐Rs dissociated from PKA‐Cs are mainly degraded by proteasome to keep the liberated state of PKA‐Cs. Increase in PKAR2β inhibits postsynaptic functions by attenuating PKA activity (Weise et al., 2018). Aβ treatment of the cultured hippocampal neurons leads to the inactivation of PKA with persistence of its regulatory subunit PKAR2α (Vitolo et al., 2002). A significantly elevated PKAR2α in the nuclear fraction with unchanged cAMP level was also shown in the present study after overexpressing hTau. Therefore, the elevation of nuclear PKAR2α may be responsible for the hTau‐induced PKA inhibition. Three major intracellular clearance systems, including the autophagic–lysosomal network (ALN), chaperone‐mediated autophagy (CMA), and the ubiquitin‐proteasome system (UPS), have all been identified in neurons. The ALN and CMA mainly function in the cytoplasm, while the UPS mainly operates in the nuclei (Boland et al., 2018). Studies also show that P301L‐tau inhibits 26S proteasome (Myeku et al., 2016). These findings suggest a link between nuclear‐elevated PKAR2α and the impaired proteasome system.

To explore the mechanism underlying hTau‐induced PKAR2α elevation and proteasome inhibition in nuclear fraction, we studied PA28γ, the nuclear activator of 20S proteasome subunit. A significantly increased PA28γ–PKAR2α interaction and decreased complex formation of PA28γ and 20S proteasome subunit with a reduced association of 20S proteasome subunit with PKAR2α were detected in hTau‐overexpressing neurons (Figure 6a–c). These data indicate that proteasome inhibition is responsible for the hTau‐induced nuclear elevation of PKAR2α and the latter mediates hTau‐induced PKA inhibition and CREB dephosphorylation. PKA increases 26S proteasome abundance and stability by promoting the association of 20S core with the 19S regulatory complex, facilitating protein degradation (Lokireddy et al., 2015). Activation of proteasome through PKA leads to proteasome‐mediated degradation in APP/PS1 mice and Tg4510 mice (Myeku et al., 2016; Smith, Pozueta, Gong, Arancio, & Shelanski, 2009). We observed that the proteasome activation by PA28γ overexpression attenuated PKAR2α elevation and CREB dephosphorylation. Recent studies have shown degradation of tau by proteasome (Boland et al., 2018; Opoku‐Nsiah & Gestwicki, 2018), while our current study provides a new mechanism of PA28γ‐20S proteasome/PKAR2α in tauopathy. Understanding the mechanism of PKA and proteasome in tauopathy is crucial for therapeutic strategies design in AD.

## 4 MATERIALS AND METHODS

### 4.1 Plasmids, viruses, reagents, and antibodies

The wild‐type full‐length human 2N4R tau (termed as hTau) was used (Goedert, Spillantini, Jakes, Rutherford, & Crowther, 1989). The EGFP‐hTau plasmid was a kind gift from Dr. Fei Liu (Jiangsu key Laboratory of neurodegeneration, Nantong University, China). The lenti‐syn‐hTau‐mCherry and AAV‐syn‐hTau‐EGFP were constructed and packaged by Obio Technology. AAV2/8 was used in animal experiments. The PA28γ overexpression virus (lenti‐syn‐PMSE3‐2A‐EGFP), PKAR2α shRNA virus (U6‐MCS‐Ubi‐EGFP‐Riiad1), and the controls were constructed by Obio Technology. PKAR2α siRNA and the control were constructed by RiboBio, and the target sequence of PKAR2α shRNA or siRNA is GACCAGAGAACATTCTTGAAT and GACTCGGATCGCAAATGAA. MG132 (a selective proteasome inhibitor) was from Tocris. Rolipram (a selective PKA agonist) was from Sigma‐Aldrich. SYBR Premix Ex Taq^TM^ II (TliRNaseH Plus) (used for real‐time PCR) was from Takara. All other reagents were from Sigma‐Aldrich. The antibodies used in the study are listed in Table 1.

**Table 1:** The list of antibodies used in the study

Antibody	Specificity/Immunogen	Host	Dilution	Catalogue number
HT7	Total/human aa 159–163	M	1:1,000 for WB	Thermo Fisher scientific, MN1000
Tau 5	Total/full‐length purified Cow Tau	M	1:1,000 for WB	Abcam, ab80579
PKAR1α	Total/human aa 1–30	R	1:1,000 for WB	Thermo Fisher scientific, PA5‐15368
PKAR2α	Total/human center region of R2	R	1:1,000 for WB 1:100 for IF	Thermo Fisher scientific, PA5‐78123
PKAR1β	Total/human aa 50–80	R	1:1,000 for WB	Thermo Fisher scientific, PA5‐13798
PKAR2β	Total/human aa 32–62	R	1:1,000 for WB 1:100 for IF	Thermo Fisher scientific, PA5‐13799
pTrKB	Phosphorylated at Y706/human	R	1:1,000 for WB	Santa Cruz Biotechnology, sc‐135645
TrKB	Total/surrounding human aa 810	R	1:1,000 for WB	Cell Signaling, 4607
PA28γ	Total/surrounding human aa 16	R	1:1,000 for WB	Cell Signaling, 2412
PA28γ	Total/mouse aa 45–147	M	1:100 for IP	Santa Cruz Biotechnology, sc‐136025
Proteasome 20S LMP2	Total/human aa 1 to the C‐terminus	R	1:10 or IP	Abcam, ab242061
PKACα	Total/human carboxyl terminal	R	1:1,000 for WB	Cell Signaling, 4782
phospho‐PKA substrate	Phosphorylated PKA substrate peptide	R	1:1,000 for WB	Cell Signaling, 9624
GluN1	Total/surrounding human aa 889	R	1:1,000 for WB	Abcam, ab68144
GluN2A	Total/mouse C terminal	R	1:1,000 for WB	Abcam, ab14596
GluN2B	Total/surrounding rat aa 1,450	R	1:1,000 for WB	Abcam, ab65783
GluA1	Total/surrounding human aa 275	R	1:1,000 for WB 1:100 for IF	Cell Signaling, 13185
pGluA1	Phosphorylated at Ser845/human	R	1:1,000 for WB	Cell Signaling, 8084
GluA2	Total/surrounding human aa 580	R	1:1,000 for WB	Cell Signaling, 13607
Synaptotagmin	Total/rat synaptic junctional protein	M	1:1,000 for WB	Abcam, ab13259
Synaptophysin	Total/human aa 250–350	R	1:1,000 for WB	Abcam, ab32127
Synapsin I	Total/rat aa 600–700	R	1:1,000 for WB	Abcam, ab64581
syntaxin	Total/rat aa 3–225	M	1:1,000 for WB	Abcam, ab13259
pCREB	Phosphorylated at Ser133/human	R	1:1,000 for WB	Cell Signaling, 9198
CREB	Total/human amino terminal	R	1:500 for WB	Cell Signaling, 9197
BDNF	Total/surrounding human aa 150	R	1:1,000 for WB	Abcam, ab108319
PKACβ	Total/human aa 1–351	R	1:1,000 for WB	Abcam, ab187515
PKACγ	Total/human C terminal	R	1:1,000 for WB	Abcam, ab108385
MAP2	Total/rat MAP2 aa 1–100	R	1:400 for IF	Abcam, ab32454
β‐actin	Total/aa 1–14	M	1:1,000 for WB	Abcam, ab6272
GAPDH	Total/rabbit muscle GAPDH	M	1:1,000 for WB	Abcam, ab8245
LaminB1	Total/mouse aa 400–500	R	1:1,000 for WB	Abcam, ab16048

Abbreviation: aa: amino acid; IF: immunofluorescence; IP: immunoprecipitation; M: mouse; R: rabbit; WB: Western blotting.

### 4.2 Animals, stereotaxic surgery, and drug treatment

Wild‐type male C57BL/6J mice (2‐month‐old, 110 ± 10 g) were purchased from Experimental Animal Center of Wuhan University and kept with accessible food and water under a 12‐hr light/dark cycle. All animal experiments were approved by the Ethics Committee of Tongji Medical College. Mice were anesthetized with isoflurane and placed on a stereotaxic apparatus and then were sterilized with iodophor, and the scalp was incised along the midline of the head. Hole was stereotaxically drilled in the skull at posterior 2.2 mm, lateral 2.6 mm, and ventral 2.3 mm relative to the bregma. Using a microinjection system (World Precision Instruments), AAV‐syn‐hTau‐EGFP or control vector (1 μl, 3.78 × 1,012 viral genome per milliliter) was injected into bilateral hippocampal CA3 region at a rate of 0.125 μl/min, the needle was kept for 10 min before withdrawal, the skin was sutured, and mice were placed beside on a heater for recovery. Rolipram was diluted to 3 mg/ml with sterile 0.9% saline containing 5% (vol/vol) Tween‐80 and 5% (vol/vol) PEG‐400. Four weeks after virus infusion, rolipram was intraperitoneally injected (0.03 mg/kg) for continuous 20 days.

### 4.3 Cell culture

N2a cells were cultured with 45% DMEM‐high glucose medium and 45% Opti‐MEM^®^ I Reduced Serum Medium and supplemented with 10% fetal bovine serum (FBS), 100 U/ml penicillin, 0.1 mg/ml streptomycin (all from Hyclone) at 37°C in the presence of 5% CO_2_. Transfection was performed with neofect (Neofect biological Technology) when cells were cultured to 70%~80% confluence in six‐well plates. 48 hr after transfection, cells were collected and lysed for further research.

For primary hippocampal neurons culture, neurons were isolated from 17 day‐ to 19 day‐embryonic Sprague Dawley rats. Hippocampus was isolated and minced into ice‐cold Hank's buffered saline solution, and incubated in 0.25% trypsin at 37°C for 15 min, then using a pipette gently triturate the tissue eight to ten times to dissociate the cells and obtain a homogenous cell suspension, and determine the density of cells on a hemocytometer. Neurons were planted with the F‐12 medium containing 10% FBS on a coverslip in a 12‐well plate with 20,000 cells per well or on a six‐well plate with 100,000 cells per well. 4 hr after planting, planting medium was gently removed and 1.5 ml of fresh maintenance medium containing 97% neurobasal medium, 2% B27, and 1% Glutamax warmed at 37°C was added into each well. Lentiviruses were used with MOI of 10 in primary hippocampal neurons at 5 *div*.

### 4.4 Western blotting and immunoprecipitation

Western blotting was performed as previously described. Briefly, N2a cells or primary neurons were collected and lysed with RIPA buffer for 10 min, and then centrifuged at 12,000 g for 10 min at 4°C. Mice were anesthetized with isoflurane and then sacrificed. The mice brain subset hippocampal CA3 were separated with vibrating microtome (Leica, VT1000S) on ice‐cold PBS according to the mouse brain atlas, and then, the CA3 tissue was homogenized brain with RIPA buffer and centrifuged at 12, 000 g for 15 min at 4°C. Then, the supernatants were collected and the protein concentration was measured using the BCA method, and loading buffer was added before electrophoresis. The protein was separated by SDS/PAGE and transferred onto nitrocellulose membranes (Whatman), then incubated with primary antibodies at 4°C overnight. Incubation of secondary antibodies was performed at room temperature for 1 hr. The membranes were developed using the ECL detection system.

For immunoprecipitation, the cultured hippocampal neurons were lysed on ice with RIPA buffer for 10 min containing 1:100 PMSF and 1:1,000 protease inhibitor cocktail (AEBSF, aprotinin, bestatin, leupeptin, E‐64, and pepstatin A), centrifuged at 12,000 g for 10 min. 200 μl of supernatants containing about 200 μg of total proteins was incubated at 4°C overnight on rotation at 4°C with 2 μg of antibody followed by the addition protein A + G agarose at 4°C for 2 hr. The agarose beads were washed three times and resuspended in 50 μl of sample buffer containing 50 mM Tris‐HCl, pH 7.6, 2% (wt/vol) SDS, 10% (vol/vol) glycerol, 10 mM DTT, 0.2% (wt/vol) bromophenol blue, and then denatured at 95°C for 10 min. Immunoprecipitants were analyzed by Western blotting with other antibodies.

### 4.5 Proteasome activity

Proteasome activity was measured using a Proteasome Activity Fluorometric Assay Kit (K245‐100, Biovision).

### 4.6 Measurement of cAMP

Monoclonal Anti‐cAMP antibody‐based direct cAMP enzyme‐linked immuno‐absorbent assay (ELISA) kit was used to measure the content of cAMP in Vec‐ and hTau‐transfected cells. Experiment was performed as described in the instruction.

### 4.7 PKA activity

PKA activity was measured by PKA kinase activity kit (Enzo Life Sciences, ADI‐EKS‐390A), which is based on a solid phase ELISA that utilizes a synthetic peptide as a substrate for PKA and a polyclonal antibody that recognizes the phosphorylated form of substrate. The absorbance was measured in a microplate reader at 450 nm.

### 4.8 Immunofluorescence

Neurons fixed in 4% paraformaldehyde for 15 min or frozen brain sections were permeabilized in 0.5% Triton X‐100 phosphate buffer for 30 min, followed by incubation with phosphate buffer containing 0.1% Triton X‐100 and 3% BSA to block nonspecific binding. The incubation of primary antibodies was performed at 4°C overnight. After washing with PBS for three times, secondary antibodies were added and incubated at 37°C for 1 hr. For labeling of surface GluA1 in living neurons, GluA1 N‐terminal antibodies (1:100) were added and incubated at 37°C for 1 hr. Neurons were then washed, fixed, and incubated with secondary antibodies. Samples were counterstained with DAPI. Images were acquired using Carl Zeiss LSM710 confocal microscope.

### 4.9 Dendrite and spine analysis

Dendritic length and complexity were analyzed by Sholl analysis using Image‐Pro Plus 6.0 software. The hippocampal neurons (5 *div*) were infected with lenti‐syn‐mCherry‐hTau or the control virus, after cultured for another 13 days, and the neurons were fixed in 4% paraformaldehyde, and then incubated with polyclone anti‐MAP2 antibody at 4°C overnight. The neurons were washed in PBS and incubated with Alexa flour 488 secondary antibody at room temperature for 1 hr. Images were captured using Carl Zeiss LSM710 confocal microscope. At least 15 cultured neurons from three different cultures were used for quantitative analysis per group by single‐blind method.

For in vitro spine analysis, primary hippocampal neurons (12 *div*) were infected with lenti‐EGFP and lenti‐syn‐hTau‐mCherry or the control vector, and the fluorescent images were obtained at 21 *div* with a confocal microscope (LSM710, Zeiss, Germany). The spine number in the dendrites per 10 mm was analyzed using an ImageJ software.

### 4.10 RT‐qPCR

Total RNA was isolated by Trizol™ (Invitrogen, CA) and reversely transcribed using the PrimeScript™ RT Master Mix (Perfect Real Time). The produced cDNA was used for real‐time PCR with primer sets: 5′‐TGTGACAGTATTAGCGAGTG‐3′ and 5′‐GCATTGCGAGTTCCAG‐3′ used for BDNF, 5′‐TCCTCGCTGTCTAACGC‐3′ and 5′‐CATGGATCTTCTTCCCTTT‐3′ used for SYN1, 5′‐CTGCCAGAAGGACCTGTATG‐3′ and 5′‐TAGCCAGAAAGTCCATCA‐3′ used for SYP, 5′‐GAGGAAAGAACGCCATTA‐3′ and 5′‐GGGCACCTTGAAAGTAAA‐3′ used for SYT, and 5′‐GTTGACATCCGTAAAGACC‐3′ and 5′‐GGAGCCAGGGCAGTAA‐3′ used for β‐actin.

### 4.11 Novel object recognition

The mice were habituated to the box (50 cm × 50 cm × 50 cm plastic container) for 5 min before the test. The box was cleaned with 70% ethanol after each mouse test. In the training trail, mice were granted 5 min to familiarize themselves with A object and B object. The box was cleaned with 70% ethanol between each trail as well. Exactly 24 hr after the familiarization period, B object was replaced with a novel C object, and the mice were granted 5 min to explore both objects. The behavior was recorded by a video camera positioned above the box. The recognition times (T) for A, B, and C objects were recorded and calculated. The recognition index (TA/(TA + TB), TB/(TA + TB) and TC/(TA + TC)) and discrimination index (TC − TA)/(TA + TC) were analyzed as described previously (Lueptow, 2017).

### 4.12 Morris water maze

The MWM test was performed from 2:00 p.m. to 8:00 p.m. as described before (Yin et al., 2016). In the learning period, three trials a day for consecutive 6 days, mice were granted in the water maze to find a hidden platform under the milky water for 60 s. In each trial, the mice started from one of the four quadrants (exclude the target quadrant) facing the wall and ended when the mice arrived on the platform. The mice were guided onto the platform if the mice did not find the platform in 60 s. The latency and the swimming path were recorded by a video camera 1.5 m from the water surface. Two days after training, the spatial memory was measured. Mice were granted to explore the water maze for 60 s with the platform removed. The time used to enter the target platform area, time spend in the target quadrant, and the number of target platform crossings were recorded by a digital video camera connected to a computer (Chengdu Taimeng Software Co. Ltd).

### 4.13 LTP recording

Mice brains were cut into horizontal sections of 400 μm thickness by a Leica VT1000s vibration microtome in ice‐cold artificial cerebrospinal fluid (aCSF) containing (mM): 126 NaCl, 3 KCl, 1.25 NaH_2_PO_4_, 24 NaHCO_3_, 2 MgSO_4_, 2 CaCl_2_, and 10 Glucose, equilibrated with 95% O_2_ and 5% CO_2_. Then, slices were transferred to a chamber filled with ACSF in a 30°C water bath and incubated for 1h. Single slice was placed on a 8*8 planar microelectrode array, with an interpolar distance of 150 μm (MED‐PA5455; Alpha MED Sciences) and kept submerged in aCSF (2 ml/min, 30°C). The fEPSP in CA1 neurons was recorded by stimulating the Schaeffer fibers from CA3. LTP was induced through applying three trains of high‐frequency stimulation (HFS, 100 Hz, 1‐s duration). Paired‐pulse facilitation was examined by applying pairs of pulse, which were separated by 50‐ to 300‐ms intervals.

### 4.14 Whole‐cell patch‐clamp recording

Primary rat hippocampal neurons (8 *div*) cultured in a dish of 35 mm diameter were infected with lenti‐syn‐hTau‐mCherry or the control vector for 7 days and then treated with rolipram for 12 hr. For whole‐cell patch‐clamp recording, culture medium was replaced with the bath solution containing (in mM): 140 NaCl, 3 KCl, 1.5 MgCl_2_, 10 HEPES, 11 Glucose, and 2.5 CaCl_2_ (pH = 7.4), which were supplemented with 1 μM tetrodotoxin and 2 μM bicuculline. The microelectrodes (3–6 MΩ) were tip‐filled with internal solution composed of 128 mM Cs‐methanesulfonate, 17.5 mM CsCl, 9 mM NaCl, 1 mM MgCl_2_, 0.2 mM EGTA, and 10 mM HEPES (pH 7.4). Cells were held at −70 mV in voltage‐clamp mode to record mEPSC. Input and series resistances were monitored continuously, and data were discarded if either of them changed more than 20%. All data were obtained using a MultiClamp 700B patch‐clamp amplifier, sampled at 10 kHz, and filtered at 2 kHz using a Digidata 1550B analog–digital interface (molecular devices). mEPSCs were analyzed using Clamp fit 10.6 (Axon Instruments).

### 4.15 Golgi staining

Golgi staining was performed by using a FD Rapid Golgi Stain Kit (FD neurotechnology, PK401). Briefly, mice were anesthetized by isoflurane and intracardially perfused with normal saline for 10 min. Brains were dissected out and immersed in an impregnation solution (equal solution A and B, containing mercuric chloride, potassium dichromate, and potassium chromate) for 24 at room temperature, impregnation solution was replaced, and samples were stored for another 2 weeks. Then, the brains were transferred to solution C for 72 hr. The brain was cut into sections of 100 μm thickness by a Leica VT1000s vibration microtome, and sections were mounted on gelatin‐coated microscope slides with solution C. Slides were rinsed twice in distilled water (2 min each) and then placed in a mixture of solution D:E:distilled water (1:1:2) for 10 min. After rinsing for distilled water, sections were dehydrated in 50%, 75%, 95%, and 100% ethanol for 4 times (5 min each). Sections were immersed in xylene (Sinopharm Chemical Reagent, 10023418) for three times (5 min each) and then mounted with cytoseal. The images were taken using Olympus BX60 (Tokyo). ImageJ software was used to analysis the spine density, which was presented as the number of spines per 10 μm of dendritic length.

### 4.16 Thioflavin‐S staining

To confirm the existence of tau fibrils, Thioflavin‐S (Sigma) staining was performed. Mouse brain slices with CA3 injected with AAV‐GFP or AAV‐hTau‐EGFP were incubated in 0.25% potassium permanganate solution for 20 min and then rinsed in distilled water and incubated in bleaching solution (containing (in distilled water): 2% oxalic acid and 1% potassium metabisulfite) for 2 min. The slices were incubated with blocking solution (containing (in water): 1% sodium hydroxide and 0.9% hydrogen peroxide) for 20 min, 0.25% acidic acid for 5 s, then washed and stained for 5 min with 0.0125% Thioflavin‐S in 50% ethanol. Then, the slices were washed with 50% ethanol for 3 times and PBS for another 3 times. Then, the slices were covered with mounting solution and inspected under a confocal microscope.

### 4.17 Statistical analysis

Data were expressed as mean ± *SEM* and analyzed by GraphPad Prism 6 statistical software (GraphPad Prism). The Student's *t* test was used for the comparison between two groups, and the one‐way ANOVA or two‐way ANOVA was used to analyze the data among three or four groups. The statistical significance was assessed at *p* < .05.

## CONFLICT OF INTEREST

The authors declare no conflict of interest.

## AUTHORS’ CONTRIBUTIONS

J.Z.W. initiated the research; J.W.Y., Y.L.Y., H.H.L., L.F., Y.Z., X.Q.T., L.Y.W., Y.Y., and D.K. performed the experiments; J.W.Y., Y.L.Y., and H.H.L. analyzed data; Y. L.Y. and J.Z.W. wrote the paper.
