# Supplementary material for: Tau inhibits PKA by nuclear proteasome‐dependent PKAR2α elevation with suppressed CREB/GluA1 phosphorylation
Source: Aging Cell. 2019 Oct 31;19(1):e13055. doi: 10.1111/acel.13055 (PMC6974714; doi:10.1111/acel.13055)
Supplement: Supplementary file 1 [file ACEL-19-e13055-s001.docx]

**SUPPOTING INFORMATION**

**Supplementary Figure 1**


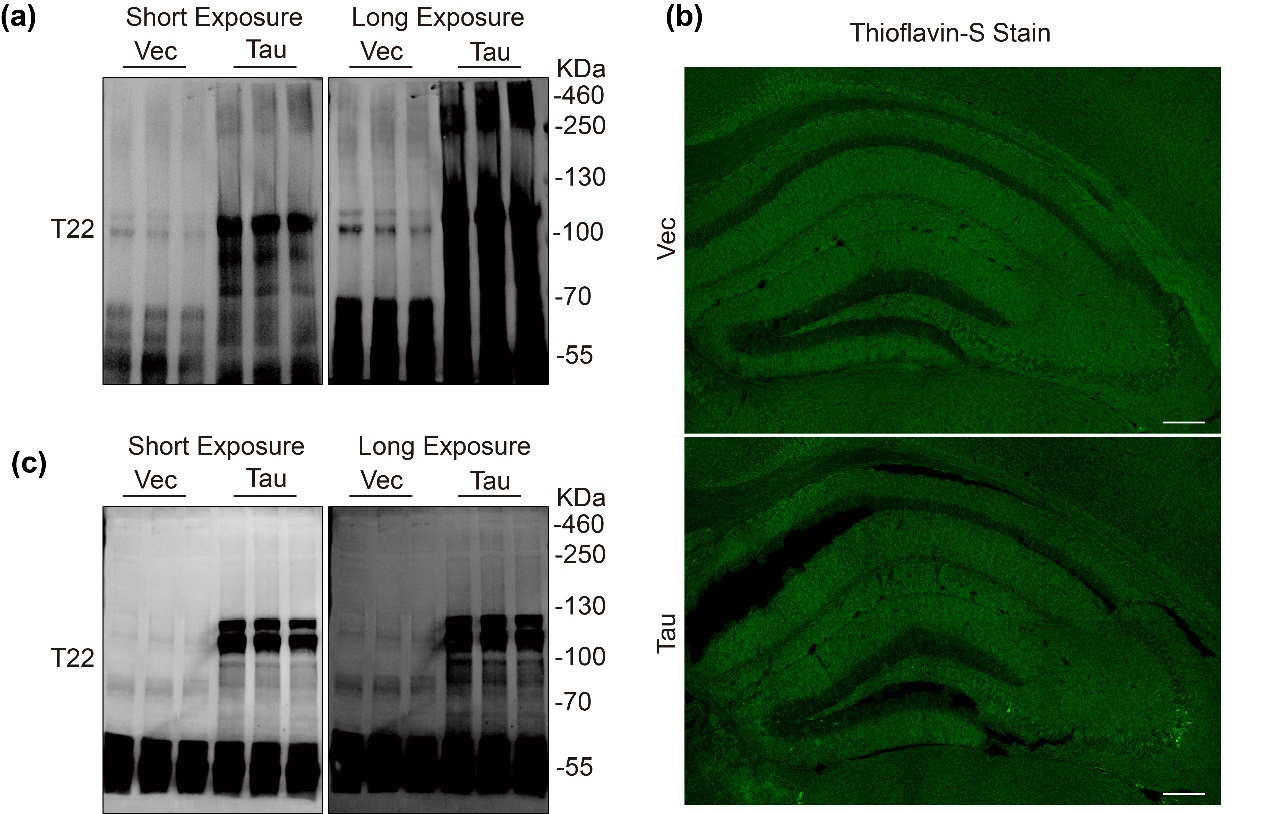


**S-Figure 1. Overexpressing hTau increases the formation of tau oligomer and fibrils *in vivo* and *in vitro*.**

(a) The AAV-hTau or the empty vector was injected in CA3 region of the hippocampus in 2-moth-old mice. After 1 month, the oligomeric tau (70~100 KDa, 130~460 KDa) in CA3 total extract were measured by Western blotting using antibody T22 (specifically reacts oligomer tau). It was observed obviously the formation of tau oligomers (70~100 KDa, 130~460 KDa).

(b) The fibrillary tau was detected by Thio-S staining. (scale bar: 200 μm).

(c) The lenti-hTau or the empty was infected in primary cultured hippocampal neurons at 5 *div*, after

7 days, the oligomeric tau was detected by T22. Weak tau oligomer bands (70~100 KDa, 130~460 KDa) were detected after only enhanced exposure.

For Western blotting the n number was 6 for CA3 extract (from 6 mice brain) and 9 for primary neurons (from 9 different batches of cultured hippocampal neurons). and for the Thio-S staining the n number was 6 (from 6 mice).

**Supplementary Figure 2**


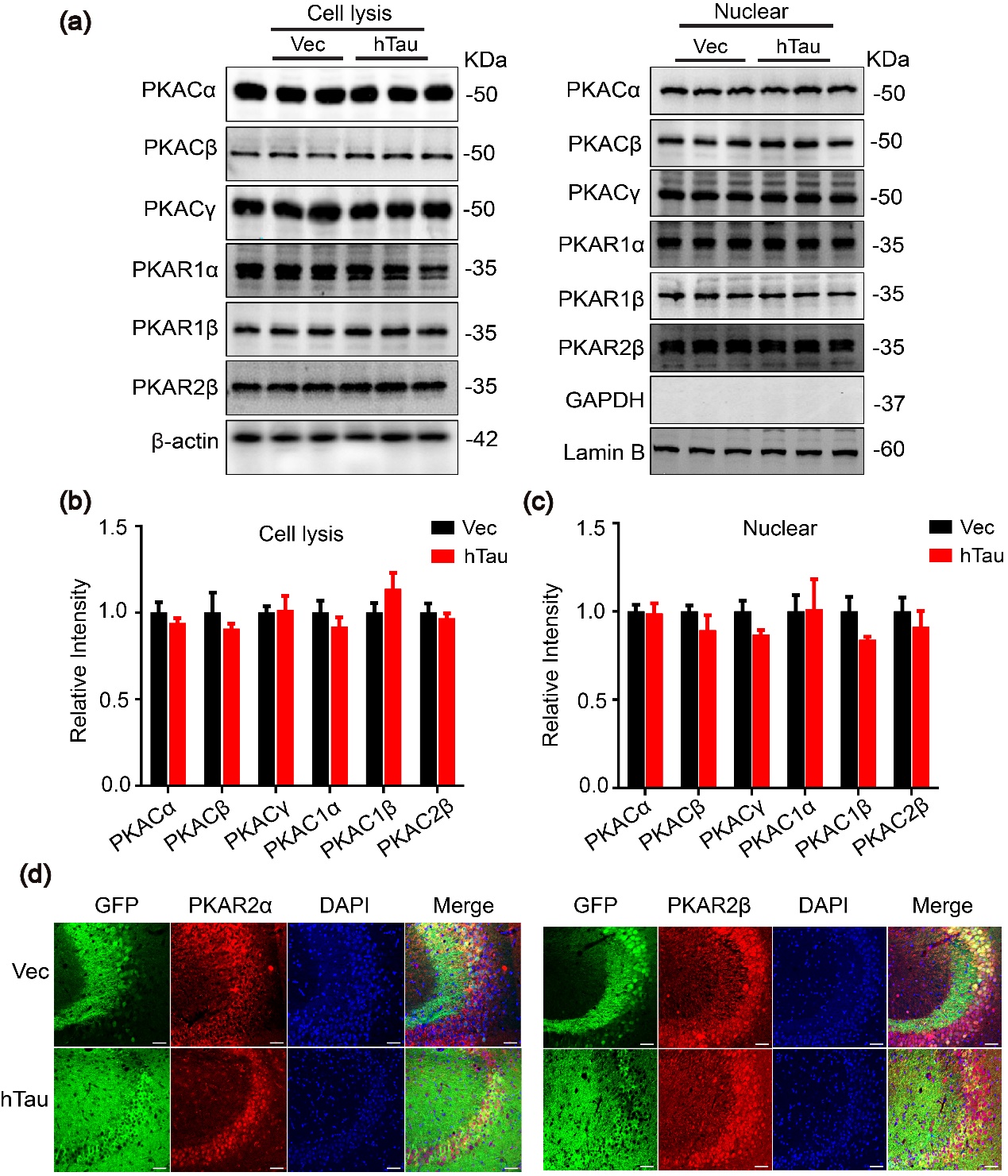


**S-Figure 2. Overexpressing hTau elevates PKAR2α in the nuclear fraction with unchanged other PKACs and PKARs.**

(a) The hippocampal neurons (*5 div*) were infected with lenti-syn-hTau-mCherry or the empty vector for 7 days, then the total cell lysis and the nuclear extract were prepared for analyzing the levels of PKACα, PKACβ, and PKACγ (PKA catalytic subunits) and PKAR1α, PKAR1β, and PKAR2β (PKA regulatory subunits). The expression of proteins from the total cell lysis (b) and the nucleus (c) were analyzed. The experiments were repeated at least for three times from 9 different batches of primary cultured neurons.

(d) Overexpressing hTau in mouse hippocampal CA3 increases nuclear staining of PKAR2α with an unchanged PKAR2β (red) measured by immunofluorescence staining (scale bar, 50 μm, from at least 6 different mice).

Data were expressed as mean ± SEM.

**Supplementary Figure 3**


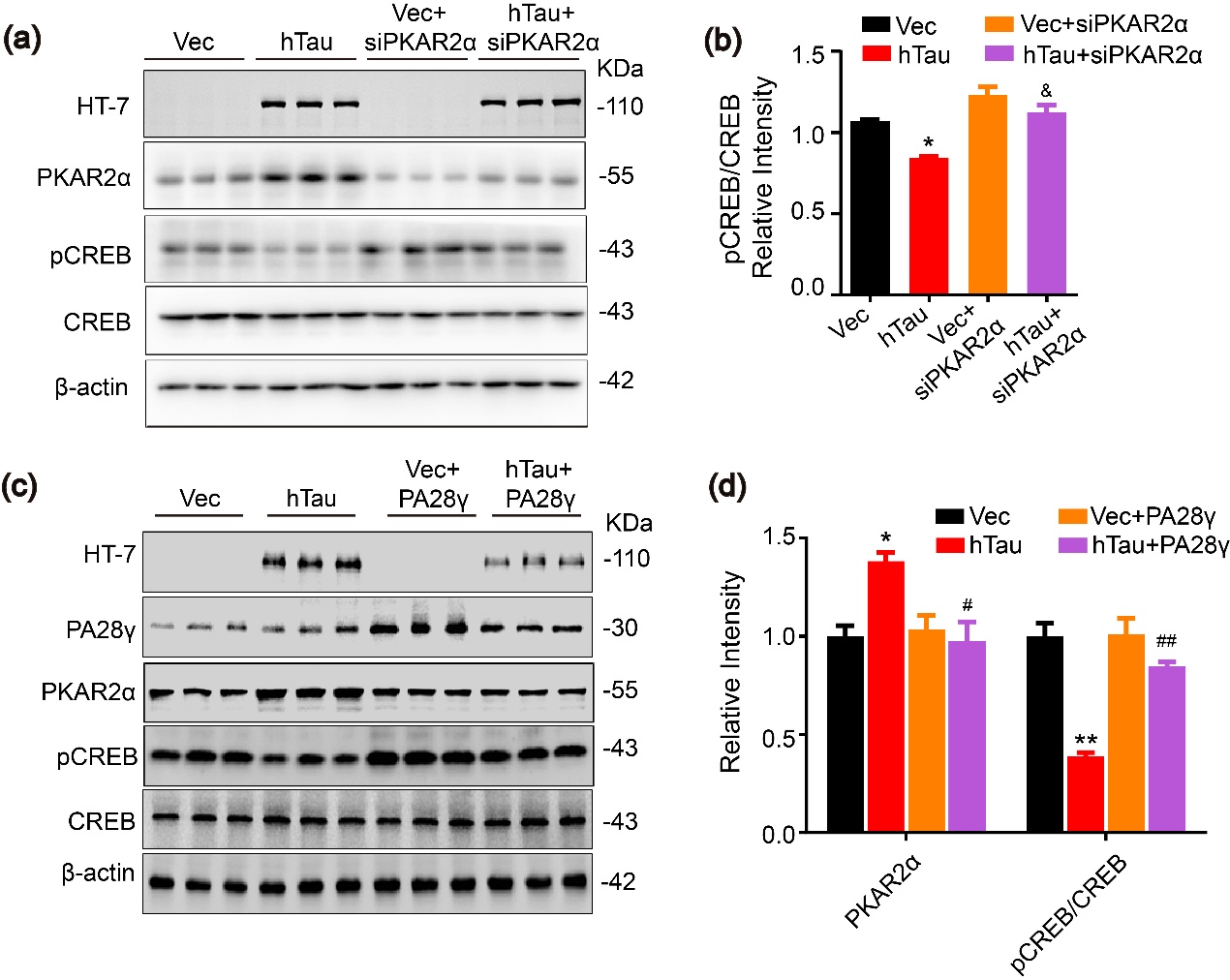


**S-Figure 3. Downregulating PKAR2α or upregulating proteasome activity attenuates hTau-induced PKA/CREB inhibition.**

(a,b) Knockdown PKAR2α by siPKAR2α attenuates hTau-induced downregulation of CREB/BDNF signaling in N2a cells co-transfected with hTau or Vec and siPKAR2α for 48 h.

(c, d) Upregulating PA28γ by co-expressing PA28γ and hTau in N2a cells for 48 h reduces hTau-induced PKAR2α elevation with restoration of CREB phosphorylation.

Data were expressed as mean ± SEM. **p* < 0.05, ***p* < 0.05 *vs.* Vec; ^&^*p* < 0.05 *vs.* hTau in (b), ^#^*p* < 0.05, ^##^*p* < 0.05 *vs.* hTau in (d). The experiments were repeated three times from 9 different batches of cells.
